# Health Tract, No. 56

**Published:** 1865

**Authors:** 


### HEALTH TRACT, No. 56.

### ERYSIPELAS.

From the two Greek words, meaning "to draw" and "neighboring;" from the nature of the disease to draw in or involve adjacent parts. The Scotch call it the "rose," from its color; others, St. Anthony's fire, from its burning heat. It is a diffused inflammation or redness of the skin of the face and head; fever precedes the local inflammation, with sore-throat as an almost invariable attendant. The premonitory symptoms are: the patient feels ill, shivery, feeble or tired, languid, and often drowsy; sometimes there is nausea, vomiting, and diarrhea. The actual attack begins with a chill; then some part of the face, nose, one cheek, or rim of one ear, begins to feel hot, stiff, and tingling, and on close examination is found to be of a deep, continuous red color, swollen and hard; this redness and swelling advances gradually, sometimes rapidly, with a distinct, elevated margin as of a wave, until the whole scalp and face are involved. No disease, except the small-pox, so obliterates and deforms the features; for the cheeks enlarge, the lips thicken enormously, and the eyes are completely closed by the swelling of the lids; the mind begins to wander, especially at night; then delirium, and in a few days, death! In cases of recovery, the redness declines in three or four days, the swelling subsides, and the person gradually gets well. Erysipelas of the head and face is so generally fatal in three or four days, spreads with such rapidity, and by extending to the throat, which by its swelling closes up the passage of the air to the lungs, causing instant death, it is important to know the distinguishing symptoms already enumerated, and to have some means at hand, by which families many miles distant from medical aid, may do something toward arresting its wave-like progress, until the physician arrives. This is, of late, claimed to be done by the very simple process of pounding raw cranberries, and covering the part affected with a poultice made of them. A more generally accessible remedy is, to paint the whole affected surface, *and a little beyond*, with common white paint, laying it on with a feather; add a fresh coat every two hours, until a thick layer is obtained, and thereafter, sufficiently often to keep the parts entirely and perfectly covered; the object being to exclude the air, which is supposed to be the great irritant. This coating of white lead paint peels off in a week or ten days with the shed skin, and leaves the surface beneath clean, smooth, and healthy. To make assurance doubly sure, promptly unload the bowels by an injection of a pint of lukewarm water; eat nothing but a crust of cold bread or toasted bread broken into some warm tea, every four hours during daylight, and occupy a clean, dry, well-aired room and bed. When the physician arrives, if you are not well, put the case implicitly and entirely into his hands. The almost universal cause of erysipelas is bad blood, arising, in nearly every instance, from constipation of the bowels; that is, their failure to act every day. This is generally brought on by resisting the calls of nature; by over-eating; by neglect of exercise in the open air, or by cooling off too quickly after such exercise; in such cases, the cold is apt to settle in the throat, and prove speedily fatal. If erysipelas sets in after a wound, it is because of the impure state of the blood from the same causes—the wound, in this case, being merely the excitant, the spark to the powder already there.

